# Development of an Improved 3D *in vitro* Intestinal Model to Perform Permeability Studies of Paracellular Compounds

**DOI:** 10.3389/fbioe.2020.524018

**Published:** 2020-09-17

**Authors:** Maria Helena Macedo, Elena Martínez, Cristina C. Barrias, Bruno Sarmento

**Affiliations:** ^1^Instituto de Investigação e Inovação em Saúde, Universidade do Porto, Porto, Portugal; ^2^Instituto de Ciências Biomédicas Abel Salazar, Universidade do Porto, Porto, Portugal; ^3^Institute for Bioengineering of Catalonia, Barcelona, Spain; ^4^Consorcio Centro de Investigación Biomédica en Red de Bioingeniería, Biomateriales y Nanomedicina, Madrid, Spain; ^5^Department of Electronics and Biomedical Engineering, Universitat de Barcelona, Barcelona, Spain; ^6^CESPU–Instituto de Investigação e Formação Avançada em Ciências e Tecnologias da Saúde, Gandra, Portugal

**Keywords:** three-dimensional (3D), intestinal model, permeability, paracellular, collagen, drug development, hydrogel, drug absorption

## Abstract

The small intestine is the primary site of drug absorption following oral administration, making paramount the proper monitoring of the absorption process. *In vitro* tools to predict intestinal absorption are particularly important in preclinical drug development since they are less laborious and cost-intensive and raise less ethical considerations compared to *in vivo* studies. The Caco-2 model is considered the gold standard of *in vitro* intestinal models regarding the prediction of absorption of orally delivered compounds. However, this model presents several drawbacks, such as the expression of tighter tight junctions, not being suitable to perform permeability of paracellular compounds. Besides, cells are representative of only one intestinal cell type, without considering the role of non-absorptive cells on the absorption pathway of drugs. In the present study, we developed a new three-dimensional (3D) intestinal model that aims to bridge the gap between *in vitro* tools and animal studies. Our 3D model comprises a collagen layer with human intestinal fibroblasts (HIFs) embedded, mimicking the intestinal lamina propria and providing 3D support for the epithelium, composed of Caco-2 cells and mucus-producing HT29-MTX cells, creating a model that can better resemble, both in terms of composition and regarding the outcomes of drug permeability when testing paracellular compounds, the human small intestine. The optimization of the collagen layer with HIFs was performed, testing different collagen concentrations and HIF seeding densities in order to avoid collagen contraction before day 14, maintaining HIF metabolically active inside the collagen disks during time in culture. HIF morphology and extracellular matrix (ECM) deposition were assessed, confirming that fibroblasts presented a normal and healthy elongated shape and secreted fibronectin and laminin, remodeling the collagen matrix. Regarding the epithelial layer, transepithelial electrical resistance (TEER) values decreased when cells were in the 3D configuration, comparing with the 2D analogs (Caco-2 and coculture of Caco-2+HT29-MTX models), becoming more similar with *in vivo* values. The permeability assay with fluorescein isothiocyanate (FITC)–Dextran 4 kDa showed that absorption in the 3D models is significantly higher than that in the 2D models, confirming the importance of using a more biorelevant model when testing the paracellular permeability of compounds.

## Introduction

The drug development field is increasingly requesting for reliable tools that can speed up the initial phases of drug development. Indeed, evaluating the absorption of compounds at an early stage of drug discovery is extremely important. Ineffective intestinal absorption allied with undesirable metabolic stability accounts for about 50% of drug failure in clinical studies (Kennedy, 1997; Li, 2001; Dahlgren and Lennernas, 2019). Understanding the absorption potential of a molecule in the beginning will allow the reduction of animal testing, besides saving time and resources.

Considering that the oral route is the most common and practical non-invasive way to administer a drug, the need for an intestinal model that can properly mimic what happens *in vivo* is unquestionable. In fact, although being the most practical route, not all drugs are suitable to be administered orally due to either toxicity or poor absorption, and this should be determined as early as possible (Billat et al., 2017).

*In vitro* cell-based intestinal models have the advantage of being simple to obtain and produce consistent results, and this is why they have been around since the 1980s (Pereira et al., 2016). Although there have been new developments and improvements to the intestinal *in vitro* models, trying to mimic as closer as possible the human intestine, the Caco-2 model is still considered the gold standard. Caco-2 cells are cultured on Transwell^®^ inserts for 21 days in order to differentiate into enterocyte-like cells, forming a polarized monolayer with a brush border membrane, microvilli, and tight junctions (TJs) (Artursson and Karlsson, 1991). Besides, these cells express some relevant influx and efflux transporters, as well as enzymes (Maubon et al., 2007). Nevertheless, this model presents several drawbacks. The fact that these cells present tighter TJs than what is observed *in vivo* makes the permeability of paracellular compounds to be underestimated (Artursson et al., 2001; Sun et al., 2008). This happens because paracellular transport refers to the transfer of substances across the epithelium through the intercellular space between the cells forming it (Edelblum and Turner, 2015). It is a transport that is unmediated and passive down a concentration gradient. This route normally allows the permeation of hydrophilic molecules (e.g., nadolol, atenolol, terbutaline), which normally are not able to permeate through the lipid membrane and may not have affinity for membrane-bound transporters being, therefore, excluded from transcellular pathway. The TJs gating the entrance to this route restrict the paracellular pathway of transport, and this is enhanced in the Caco-2 model, where the presence of TJs is higher than in the human small intestine, forming a very tight monolayer and impeding the passage of paracellular compounds (Lennernäs et al., 1996; Artursson et al., 2001). Besides the tightness of the monolayer, the lower permeability of paracellular compounds in the Caco-2 model may also be attributed to the lower number of pores per cm^2^ that is observed in this model comparing to that of the human jejunum (Linnankoski et al., 2010).

In fact, the Caco-2 model is better at predicting the permeability of transcellular compounds than paracellular, where, normally, values of permeability are much lower than what is observed *in vivo* (Tavelin et al., 2003). To overcome these issues, other cell lines have been proposed to be used in *in vitro* intestinal models, such as Madin-Darby canine kidney (MDCK), Lewis lung carcinoma–porcine kidney 1 (LLC-PK1), 2/4/A1, TC-7 (a Caco-2 subclone), and IEC-18 (Gres et al., 1998; Marano et al., 2001; Balimane and Chong, 2005; Lazorova et al., 2011; Turco et al., 2011). Besides presenting some advantages, they all possess their own limitations, making the Caco-2 *in vitro* model the most widely accepted until today. An enhancement of the Caco-2 model has been achieved, coculturing these cells with HT29-MTX cells. These cells are known to be mucus producers, mimicking the goblet cells of the small intestine. Besides, these cells present looser TJs, decreasing the tightness of the monolayer and making it more similar to what is observed *in vivo* (Antunes et al., 2013). More recently, a triple coculture model has been developed with the addition of Raji B monocytes that are capable of secreting factors that make a fraction of Caco-2 cells differentiate into M-cells that have a role in transepithelial transport (Antunes et al., 2013). Although this model is more similar to the human small intestine and provides better results regarding the permeability of compounds, it still continues to represent only one layer of the intestine—the epithelium. Besides, cells are seeded on top of the Transwell^®^ insert membranes that do not provide biological cues to the epithelial cells and do not replicate the architecture and mechanisms occurring in the living organ. In fact, cells that are grown in two-dimensional (2D) lack the interactions that occur in *in vivo* 3D matrixes, which are crucial for maintaining cell functions and polarity (Darnell et al., 2011; Desrochers et al., 2014). The presence of a 3D matrix that can resemble the *in vivo* extracellular matrix (ECM) is of paramount importance regarding cell behavior and differentiation (Pampaloni et al., 2007; Justice et al., 2009; Chitcholtan et al., 2013). Besides, regarding intestinal absorption, the epithelium represents an important role in the absorption process, but it is not the unique anatomic barrier that conditions the permeability of drugs. The small intestine is divided into four main layers, mucosa, submucosa, muscularis propria, and serosa, although in most of the duodenum, the outer layer is the adventitia (Seeley et al., 2004; Pereira et al., 2016). The absorption process occurs in the mucosa, the most complex layer, which comprises the epithelium, lamina propria, and muscularis mucosae (Pampaloni et al., 2007). The lamina propria, which provides vascular support for the epithelium and often contains mucosal glands, like Brunner’s glands in the duodenum, which are spiral tubes that form a network in the submucosa and open through the lamina propria into the crypts between the villi, secreting a thin and alkaline mucus that helps protect the duodenum, aiding in acid neutralization (Cullen, 2008), can have a role in the absorption process and influence the behavior of the epithelial cells (Shaker and Rubin, 2010). The lamina propria is composed of connective tissue, like collagen and elastin, fibroblasts, blood, and lymphatic vessels. Nevertheless, a distinctive characteristic of this layer is that it contains a great number of immunologically competent cells, like mononuclear cells, plasma cells and lymphocytes, and nerve endings (Boudry et al., 2004). Trying to replicate the 3D intestinal environment and develop models that can better replicate the *in vivo* situation to better predict permeability outcomes is of extreme importance. Although being a relatively new topic, 3D models have been explored in the past years, and different models have been developed by different groups (Sung et al., 2011; Yu et al., 2012; Li et al., 2013; Pereira et al., 2015; Dosh et al., 2017; Yi et al., 2017; Madden et al., 2018). Each model has its own peculiarities, and there is a variety of models—from the ones that are based on scaffolds, to decellularized tissue models, to more complex gut-on-a-chip models or even organoids. Within the models that are based on scaffolds that serve as the 3D support for the cells, some are built in order to mimic the architecture of the human small intestine, namely, the villi, and authors observe that the architecture can give important cues for cells and alter their behavior *in vitro*, making cells respond to the shape and have a more physiological behavior, which can be important for several studies (Sung et al., 2011; Yu et al., 2012, 2014; Kim and Kim, 2018; Castano et al., 2019). However, it is important to consider that other aspects, besides the 3D structure and architecture, can influence the behavior of intestinal cells. For example, it has been shown that nutrients can modulate intestinal cell function and morphology, being that an excess of nutrients can lead to an increased number of epithelial cells, an increased proliferation rate, villi length and crypt depth, and even redistribution of TJ proteins (Altmann and Leblond, 1970; Brun et al., 2007; Verdam et al., 2011; Mao et al., 2013; Mah et al., 2014). These alterations were proven to occur in a Caco-2/HT29 model, where excess nutrients led to an increase in follicle-like structures and mucus production and a decrease in TJs, associated with an increase of paracellular permeability (Bottani et al., 2019). The activity of intestinal markers such as alkaline phosphatase, aminopeptidase N, and dipeptidyl peptidase-IV was raised. Besides nutrient exposure, it is also possible to modulate Caco-2 cell differentiation using different cell passages. Ferraretto et al. (2007) showed that early passages of Caco-2 cells are representative of an undifferentiated phenotype, with low enzyme activity, whether at intermediate passages (18–25) a differentiated phenotype is observed and is characterized by the presence of a mature brush border with microvilli and high enzymatic activity. From passage 26 on, the presence of microvilli is accompanied by the appearance of a junctional apparatus, which confirms the polarity and the transport functions of the monolayer.

**FIGURE 1:**
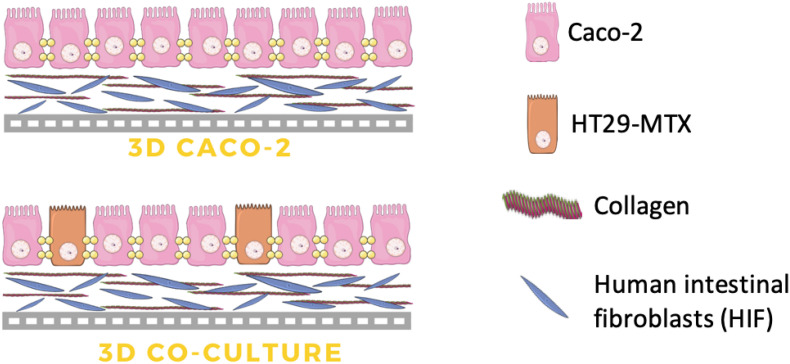
Scheme of the configuration of the three-dimensional (3D) intestinal model (3D Caco-2 and 3D Coculture). The 3D model is composed of a collagen layer with human intestinal fibroblasts embedded, and on top, Caco-2 and HT29-MTX epithelial cells are added in order to mimic the intestinal enterocytes and goblet cells, respectively.

Regarding the 3D models, not only the architecture may play an important role. The truth is that the 3D support itself, which is normally composed of ECM components, can serve as a more physiological support than the plastic membranes that are normally used and have in impact in cell behavior. In fact, not all laboratories and researchers are equipped and have the know-how to produce 3D complex structures using different techniques. So, if the aim is to develop a 3D model that can better represent the human small intestine but, at the same time, be simple enough so others can replicate it to test their compounds, maybe the complex 3D structures need to be left out. Since our aim was to develop a new model to be used for permeability testing, we opted by building a model that, although complex, could be simple enough so others could reproduce it. In this study, we propose a model that comprises a layer composed of collagen with fibroblasts embedded, which mimics the intestinal lamina propria, giving support to the epithelium and having a role in the behavior of epithelial cells and in the permeability outcomes (Figure 1). In this study, the aim was to obtain a model that could better resemble the human small intestine, making cells feel a more physiological environment and behaving in a way that could resemble more what is observed *in vivo*. Regarding the permeability of paracellular compounds, underestimated in the Caco-2 models, our hypothesis laid on the possibility of this model providing closer biomimetic conditions, considering the aforementioned reasons.

## Materials and Methods

### Cell Culture Conditions

Human colon adenocarcinoma Caco-2 cells (passage 26) were purchased from American Type Culture Collection (ATCC, United States). Mucus-producing HT29-MTX (passage 41) cells were kindly provided by Dr. T. Lesuffleur (INSERM U178, Villejuif, France). Caco-2 and HT29-MTX cell lines were grown in Dulbecco’s modified Eagle’s medium (DMEM) with 4.5 g/L glucose and Ultraglutamine^TM^ (Gibco), supplemented with 1% non-essential amino acids (NEAA) 100X (Gibco), 1% penicillin/streptomycin 100X (Biowest), and 10% fetal bovine serum (FBS) (Biochrom). Human intestinal fibroblast (HIF) primary cells (passage 6) were obtained from ScienCell and were cultured in Fibroblast Medium (FM) supplemented with 2% FBS, 1% of Fibroblast Growth Supplement (FGS), and 1% penicillin/streptomycin solution (all from ScienCell). Cells were grown separately in tissue culture flasks (SPL) and maintained in an incubator (Binder) at 37°C and 5% CO_2_ in a water-saturated atmosphere.

### Collagen Hydrogel With Human Intestinal Fibroblasts Embedded

Different initial seeding densities of HIFs were used in order to understand the appropriate cell concentration without compromising their viability. To obtain the collagen layer with HIF, 10X phosphate buffered saline (PBS), 1N sodium hydroxide (NaOH), and FM with HIF in different concentrations ranging from 1 × 10^5^ to 2 × 10^6^ cells/ml were added to the high concentrated rat tail collagen solution (Corning), following supplier recommendations, in order to obtain a final concentration of 5 mg/ml of collagen. The solution was placed on the 12-well Transwell^®^ inserts (Millicell) using 110 μl to obtain a layer with approximately 1 mm of thickness. The solution was dispersed carefully in order to cover the entire surface of the insert and was incubated at 37°C, 5% CO_2_ in a water-saturated atmosphere for 30 min to allow gelation. Disks were monitored for 21 days. Since with these conditions, contraction of the disks was observed, an increase in collagen concentration was tested. New disks with a collagen concentration of 6 mg/ml were obtained, using the same method, and HIFs were embedded using the lower density previously tested (1 × 10^5^ cells/ml). This setup was used for further experiments.

### Determination of Collagen Hydrogels Rheological Properties

To determine the rheological properties of the collagen hydrogels, the rotational rheometer Kinexus Pro (Malvern Instruments) with an 8-mm parallel disk geometry was used. Frequency sweep experiments were performed at 1% strain and a frequency ranging from 0.1 to 10 Hz within the linear viscoelastic region to determine the shear elastic and viscous moduli of the samples. The experiments were performed on collagen disks obtained as previously mentioned, without fibroblasts embedded, with approximately 1-mm thickness and cut into 8-mm diameter disks.

### Visualization of Collagen Hydrogels

Collagen disks with a concentration of 6 mg/ml, without HIFs embedded, were obtained, as mentioned previously, to visualize the fibers through atomic force microscopy (AFM) and scanning electron cryomicroscopy (CryoSEM).

Atomic force microscopy images in collagen were obtained using a PicoPlus 5500 controller (Keysight Technologies, United States). The images were performed in Contact mode, in liquid (PBS), using a V-shaped MLCT-BIO-DC-D cantilever with a spring constant (k) in the range of 0.03 N/m (Bruker Corporation, United States).

The scan speed was set at 0.5 l/s. The scan size was 30 μm^2^ × 30 μm^2^. The software used to obtain the images was the PicoView 1.2 (Keysight Technologies, United States).

The CryoSEM examination was performed using the high-resolution scanning electron microscope JSM 6301F (JEOL), X-ray microanalysis using INCA Energy 350 (Oxford), and the low-temperature sample observation system Alto-2500 (Gatan). The sample was rapidly cooled through immersion in slush nitrogen and transferred, in vacuum, to the sample preparation chamber with cooled platinum. The sample was fractured, sublimated for 120 s at −90°C and coated with Au/Pd by ionic pulverization for 50 s. The sample was then transferred to the SEM chamber, and visualization of the sample was performed at −150°C.

### Metabolic Activity of the Human Intestinal Fibroblasts Inside the Collagen Hydrogels

The metabolic activity of the HIFs inside the collagen disks was determined at different time points using the resazurin assay. Collagen hydrogels with a final concentration of 6 mg/ml and 1 × 10^5^ HIF/ml embedded were obtained, dispersed in 48-well plates, and allowed to gel for 30 min inside the incubator at 37°C, 5% CO_2_ in a water-saturated atmosphere. After complete gelation, 2 ml of DMEM was added to each well, the plate was placed again in the incubator, and the medium was changed every 2–3 days. The resazurin solution was obtained dissolving 50 mg of resazurin sodium salt (Sigma) into 25 ml of PBS 20X and 475 ml of dH_2_O overnight, at room temperature with agitation in the dark, and then filtered. At each time point (collagen disks with 1, 7, 14, and 21 days in culture), a solution containing 20% resazurin in DMEM complete was prepared. The solution was pre-warmed to 37°C, and 600 μl was added to each well. The plate was incubated for 2 h at 37°C, 5% CO_2_ in a water-saturated atmosphere in order to allow viable cells with active metabolism to reduce resazurin into the resorufin product, which is pink and fluorescent. After the incubation period, samples were quantified by measuring the relative fluorescence units (RFUs) using a microplate reader Synergy^TM^ Mx HM550 (Biotek) set at 530/590 (excitation/emission wavelength, respectively), and results were normalized by subtracting the blank (collagen gel without cells).

### Three-Dimensional *in vitro* Model

Collagen hydrogels with a concentration of 6 mg/ml and 1 × 10^5^ HIF/ml were obtained as previously described. After the 30-min gelation process, Caco-2 cells (1 × 10^5^ cells/cm^2^) (3D Caco-2) or a mixture of Caco-2 and HT29-MTX (1 × 10^5^ cells/cm^2^ at a 9:1 ratio, respectively) (3D Co-culture) in 0.5 ml of DMEM were added on top of the fibroblast-embedded collagen layer. Then, 1.5 ml of DMEM was added to the basolateral side, and plates were incubated at 37°C, 5% CO_2_ in a water-saturated atmosphere. Same cell numbers of Caco-2 (2D Caco-2) and Caco-2+HT29-MTX (2D Coculture) were added directly on top of inserts without collagen, with the same DMEM volume to use as 2D controls. The cultures were maintained during 14 or 21 days, and the medium of the models was changed every 2–3 days. Transepithelial electrical resistance (TEER) was periodically measured using an EVOM^2^ equipment (World Precision Instruments).

### Assessment of Human Intestinal Fibroblast Morphology and Extracellular Matrix Secretion

The morphology of the HIFs seeded inside the collagen hydrogels after 14 days was assessed using an antibody against vimentin, which is a filament protein present in mesenchymal cells (Danielsson et al., 2018). The expression of fibronectin and laminin, which are components of the ECM, was also assessed.

Samples were washed once with PBS and fixed using 2% paraformaldehyde (PFA) in PBS for 30 min at room temperature (RT). After the fixation step, samples were washed three times for 5 min with PBS. This step was followed by permeabilization with a solution of 0.2% (v/v) Triton X-100 in PBS for 10 min at RT, and samples were washed thrice with PBS for 5 min. Afterward, a blocking step was performed using a blocking solution (BS) containing 1.5% bovine serum albumin (BSA) and 5% FBS in PBS for 1 h at RT. Samples were then transferred to a humidified chamber and were incubated either with mouse anti-human vimentin primary antibody (1:50) (sc-6260, Santa Cruz Biotechnology) + rabbit anti-human fibronectin primary antibody (1:50) (F3648, Sigma Aldrich) or with mouse anti-human vimentin primary antibody (1:50) + rabbit anti-human laminin primary antibody (1:30) (L9393, Sigma Aldrich) overnight at 4°C. After incubation with the primary antibodies, samples were washed three times for 5 min with a solution of 0.05% Tween-20 in PBS (PBST). This was followed by incubation with the secondary antibodies F(ab’)2-Goat anti-Mouse IgG (H+L) Cross-Adsorbed, Alexa Fluor 594 (A-11020, Thermo Fisher Scientific) (1:500) + Goat anti-Rabbit IgG (H+L) Cross-Adsorbed, Alexa Fluor 488 (A-11008, Thermo Fisher Scientific) (1:500), and cell nuclei were counterstained with 4’,6-diamidino-2-phenylindole (DAPI) (Sigma Aldrich) (500 ng/ml). Finally, cells were washed twice with PBST and once with PBS for 5 min and kept in PBS until visualization by a spectral confocal laser scanning microscope TCS-SP5 AOBS (Leica).

### Assessment of Cell Layer Formation by Hematoxylin and Eosin Staining

To assess cell layer formation in both 2D models at days 14 and 21 and 3D models at day 14, hematoxylin and eosin (H&E) staining was performed. For this, inserts with the cells were washed once with PBS for 5 min and fixed using 2.5% glutaraldehyde and 2% PFA in 0.1 M phosphate buffer (PB) for 1 h at RT. Then, inserts were washed thrice with PBS for 5 min and kept in PBS at 4°C until further processing for paraffin embedding. Sections of 3 μm were obtained using an RM2255 microtome (Leica). Sections were then stained for H&E. Briefly, sections were deparaffinized and rehydrated, stained for 3 min in Gil’s Hematoxylin (Thermo Scientific), 6 min in running water, dehydrated, stained for 1 min in Eosin Y (Thermo Scientific), cleared, and mounted in Entellan (Merck).

### Assessment of Cell Layer Formation, Tight Junction, and MUC2 Expression by Immunocytochemistry

The formation of an intact layer was confirmed using an antibody against EpCAM, and the presence of TJs was assessed using claudin-1 and zona occludens 1 (ZO-1), which are integral transmembrane and peripheral TJ proteins, respectively. The presence of adherens junctions was evaluated using an antibody against *E*-chaderin. MUC2 was chosen to evaluate mucin expression because it is the mostly expressed mucin in the small intestinte (Schneider et al., 2018).

The models were washed with PBS and fixed with 2% PFA for 30 min. Then, cells were washed three times with PBS. The models were submerged in a solution of sucrose 15% (in PBS) for 3 h, and after that, they were left overnight in a solution of 30% sucrose. Then, models were embedded in Optimal Cutting Temperature (OCT) compound (Kaltek) and frozen at −20°C. Sections with 7 μm were obtained using an HM550 cryostat (Microm).

To stain with different antibodies, the slides with the cuts were dipped into PBS to dissolve the OCT. A permeabilization step using a solution of 0.2% (v/v) Triton X-100 in PBS for 5 min at RT was performed. Afterward, slides were washed thrice with PBS for 5 min. A blocking step was performed using a solution containing 1.5% BSA and 5% FBS in PBS for 1 h at RT. Slides were incubated with the primary antibodies’ solutions [rabbit anti-human EpCAM primary antibody (1:250) (10694-R028, Sino Biological) + mouse anti-human vimentin primary antibody (1:100) (sc-6260, Santa Cruz Biotechnology) or rabbit anti-human ZO-1 primary antibody (1:50) (sc-10804, Santa Cruz Biotechnology) + mouse anti-human MUC2 primary antibody (1:50) (ab118964, Abcam) or rabbit anti-human claudin-1 primary antibody (1:200) (MA5-16351, Thermo Fisher) or mouse anti-human *E*-cadherin primary antibody conjugated with Alexa Fluor 488 (1:100) (324110, Biolegend)] overnight at 4°C. Slides where washed three times for 5 min with PBST and incubated with the secondary antibodies and DAPI to counterstain the nuclei (500 ng/ml) (Sigma). For the first two combinations, F(ab’)2-Goat anti-Mouse IgG (H+L) Cross-Adsorbed, Alexa Fluor 594 (A-11020, Thermo Fisher Scientific) (1:500) and Goat anti-Rabbit IgG (H+L) Cross-Adsorbed, Alexa Fluor 488 (A-11008, Thermo Fisher Scientific) (1:500) were used, and for claudin-1, the secondary antibody against rabbit (Alexa Fluor 488) was used. Secondary antibodies were added to samples for 3 h at 4°C. For *E*-cadherin, no secondary antibody was needed, since the primary antibody was already conjugated to a fluorochrome. Slides were then washed twice with PBST and once with PBS, all for 5 min. Slides were mounted using fluorescent mounting medium (Dako). Samples were left to dry overnight protected from light at 4°C and were maintained in these conditions until visualization by a spectral confocal laser scanning microscope TCS-SP5 AOBS (Leica). Since the aim was to compare the amount and localization of tight and adherens junctions between samples, laser power and gain of each laser were maintained for all samples regarding each antibody.

### Permeability Assay With Fluorescein Isothiocyanate–Dextran

Permeability of fluorescein isothiocyanate (FITC)–Dextran 4 kDa (Sigma) was performed through the 3D and 2D intestinal models to assess the robustness of each model regarding paracellular transport. FITC-Dextran with a molecular weight of 4 kDa was chosen because it represents a measure for paracellular permeability of the intestinal epithelium (Woting and Blaut, 2018). The permeability assays were performed at the 14th and 21st day of culture.

Culture medium was removed from both sides of the Transwell^®^ inserts and washed twice with pre-warmed Hank’s balanced salt solution (HBSS). After the washes, 1.5 and 0.5 ml of HBSS were placed on the basolateral and apical sides of the inserts, respectively, and they were allowed to equilibrate for 30 min at 37°C and 100 rpm in a KS 4000 ic control orbital shaker (IKA). After 30 min, HBSS on the apical side was replaced by 0.5 ml of 200 μg/ml FITC-Dextran dissolved in HBSS. At predetermined time points (15, 30, 45, 60, 90, 180, 240, and 300 min), TEER was measured in order to assess the monolayer integrity, and a sample of 200 μl was taken from the basolateral side and replaced by the same amount of HBSS. At the end of the assay, a sample from the apical side was taken. The amount of FITC-Dextran was measured using a Synergy microplate reader (Biotek) at excitation/emission wavelengths of 490/520. The permeability results were expressed in percentage of release and apparent permeability (P_*app*_) that was calculated using the following equation:

(1) $$P_{a\,p\,p}=\frac{\Delta \,Q}{A\times C_{0}\times \Delta \,t}\,§$$

where Δ*Q* is the amount of compound detected in the basolateral side (mg), A is the surface area of the insert (cm^2^), *C*_*0*_ is the initial concentration in the apical compartment (mg/ml), and Δ*t* is the time of the experiment (s).

### Statistical Analysis

To perform statistical analysis, the software GraphPad Prism 7.0 (GraphPad Software Inc.) was used. All results are represented as mean ± standard error deviation (SEM). Differences between groups in percentage of permeability were compared using one-way analysis of variance (ANOVA) Tukey’s *post hoc* test. Differences in resazurin and Papp results were compared using *T*-student test. The level of significance was set at probabilities of ^∗^*p* < 0.05, ^∗∗^*p* < 0.01, ^∗∗∗^*p* < 0.001, and ^****^p < 0.0001.

## Results

### Contraction of the Collagen Matrix by Fibroblasts

Using the initial collagen concentration of 5 mg/ml, contraction of the disks by the fibroblasts was observed in all conditions, being that contraction was proportional to the density of cells inside the disk (Supplementary Material).

It was observed that an increase in collagen concentration to 6 mg/ml was enough to prevent collagen contraction, at least until day 14 in culture. In fact, when fibroblasts were seeded inside the collagen disks and no epithelial cells were seeded on top, disks would not contract for 20–21 days. However, when only Caco-2 cells were seeded on top of the gels, contraction of the disks occurred around day 16. Although contraction did not occur for the 3D coculture, in order to be coherent, both 3D models were used after 14 days.

### Collagen Hydrogels Properties

**FIGURE 2:**
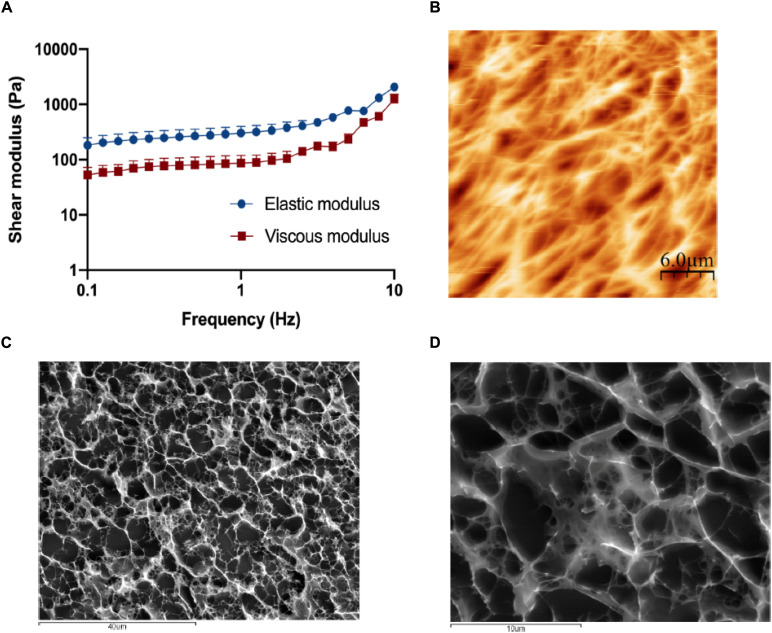
Characterization of the collagen matrix. **(A)** Rheological behavior of the 6 mg/ml collagen hydrogels when subjected to frequency sweep tests (0.1–10 Hz) with a strain of 1% show that the elastic modulus of the disks is around 500 Pa, which is considered within normal lamina propria values. Results are the average of triplicates, and bars represent the standard error deviation (SEM). **(B)** Visualization of the 6 mg/ml collagen hydrogel surface by atomic force microscopy (AFM). Collagen gels present a highly porous surface, with porous sizes ranging from 1 to 10 μm. **(C,D)** Visualization of the 6 mg/ml collagen hydrogel inner structure by CryoSEM. Like its surface, the inner structure of the disks is highly porous, with random distribution and a size ranging from 1 to 10 μm.

Rheological assays allowed the determination of the elastic and viscous components of the shear modulus (Figure 2A) under test conditions. The average value of the elastic component of the shear modulus was 483 ± 106 Pa, while the viscous component was 198 ± 18 Pa.

AFM (Figure 2B) and CryoSEM (Figures 2C,D) techniques showed that hydrogels have a porous mesh, with random porous size, ranging from less than 1–10 μm, and random distribution.

### Metabolic Activity of Human Intestinal Fibroblasts Inside the Disks

**FIGURE 3:**
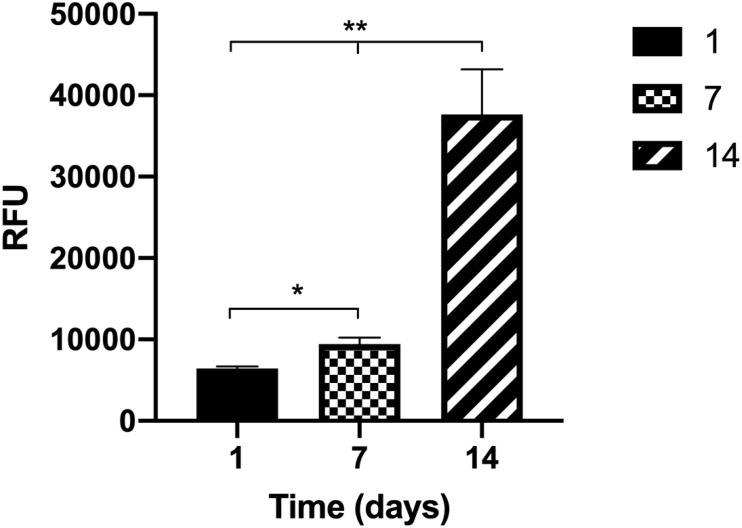
The metabolic activity of the human intestinal fibroblasts (HIFs) inside the collagen hydrogels for 14 days shows that cells have an increase in metabolic activity from week to week. Results are the average of triplicates, and bars represent the standard error deviation (SEM). Statistical differences *p* < 0.05 are denoted by * and *p* < 0.01 are denoted by **.

The metabolic activity of the fibroblasts inside the collagen disks was assessed, presenting a stairway profile along the time in culture and showing a significant increase from week to week (Figure 3). This means that fibroblasts are active inside the gels, which indicates that they are healthy and proliferating.

### Human Intestinal Fibroblast Morphology and Fibronectin and Laminin Secretion Inside the Collagen Disks

**FIGURE 4:**
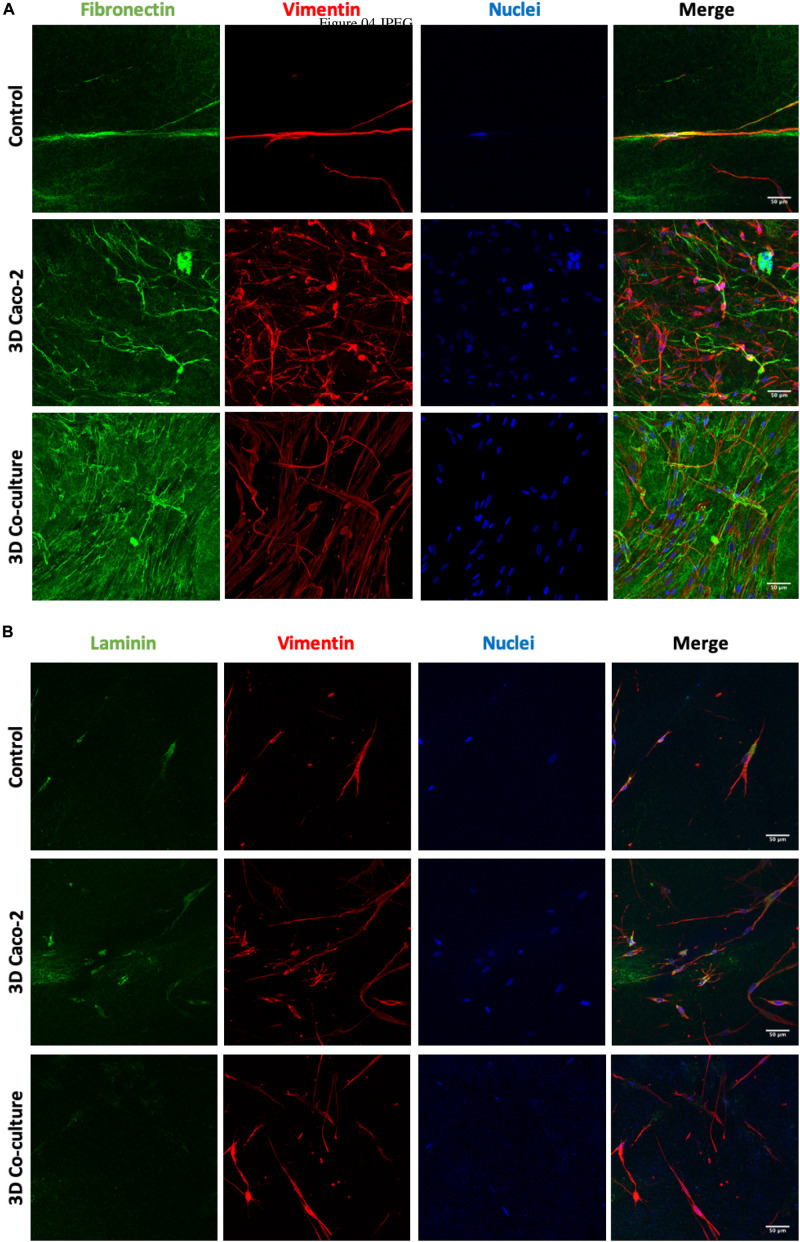
Human intestinal fibroblast (HIF) morphology and **(A)** fibronectin and **(B)** laminin deposition by HIFs in collagen hydrogels, during the time in culture, with and without epithelial cells on top. The control refers to HIF embedded in the collagen layer alone, and the 3D Caco-2 and 3D Coculture refer to the addition of Caco-2 and Caco-2 + HT29-MTX on top of this layer, respectively. Pictures show maximal projections of several stacks of the gels. Fibronectin and laminin were labeled with Alexa-Fluor 488 (green), and vimentin was labeled with Alexa-Fluor 594 (red). The nucleus was counterstained with 4′,6-diamidino-2-phenylindole (DAPI) (blue).

To observe the morphology of the HIFs inside the collagen hydrogels and understand if they present a normal morphology, as well as to assess the secretion of fibronectin and laminin, immunocytochemistry technique was used. Figure 4 depicts the elongated shape of fibroblasts in each condition. Besides, a higher cellular density and a higher amount of fibronectin and laminin (Figures 4A,B) are observed when Caco-2 or Caco-2+HT29-MTX cells are seeded on top of the gels when comparing to the disks embedded with HIFs without epithelial cells on top. Comparing the 3D Caco-2 with the 3D Coculture model, it was possible to observe that there is a higher number of fibroblasts and fibronectin deposition inside the disks when only Caco-2 cells are on top comparing to the Coculture.

### Cell Layer Formation and Expression of Proteins of the Junctions

**FIGURE 5:**
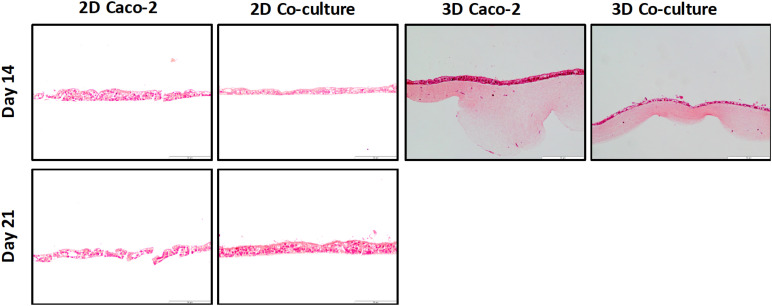
H&E staining of the different models at 14 and 21 days shows that at day 14, a cell layer is already formed in all the models.

**FIGURE 6:**
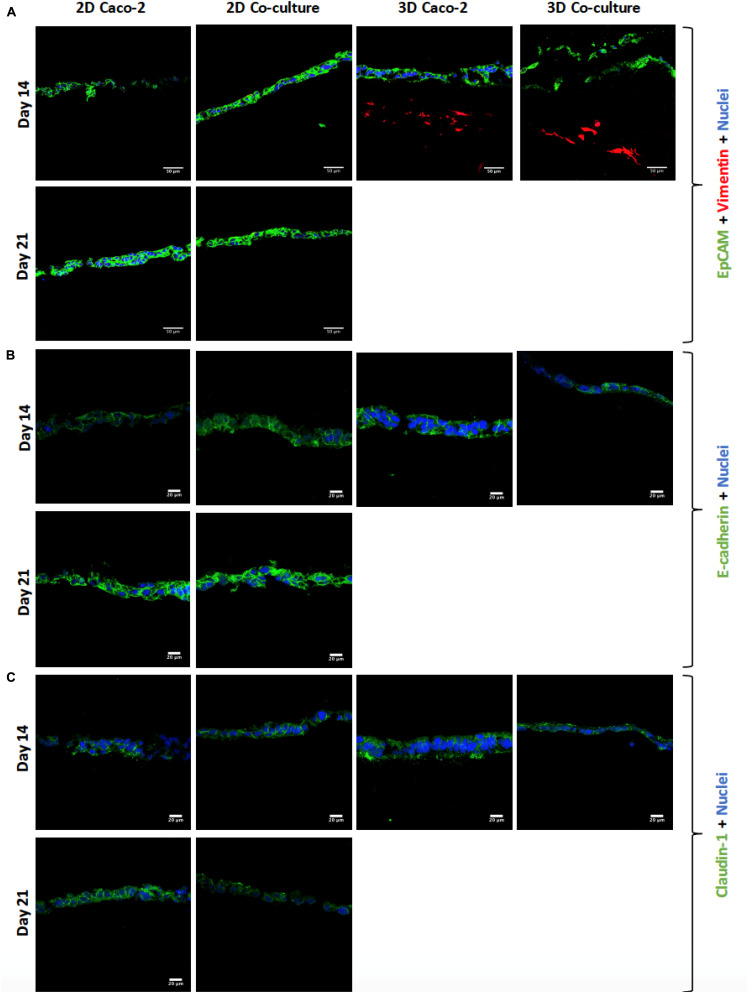
**(A)** Visualization of cell layer formation by EpCAM, confirming the presence of a cell layer in all models. **(B)** Assessment and comparison of cellular adherens junctions in different models at different time points using *E*-cadherin. **(C)** Assessment and comparison of the tight junctions’ protein claudin-1 in the different models at different time points. EpCAM, *E*-cadherin, and claudin-1 were labeled with Alexa-Fluor 488 (green), and nuclei were stained with 4′,6-diamidino-2-phenylindole (DAPI) (blue).

The cell layer formation was assessed through H&E staining and immunocytochemistry on the different time points to understand if there were differences regarding the epithelial layer in different days. It was observed that, in all models, the cellular layer is already formed at day 14 (Figures 5, 6A). It is also possible to observe some multilayer formation in the Caco-2 models, both in 2D and 3D, and these multilayers seem to be decreased in the Coculture models.

Regarding the expression of tight and adherens junctions and the secretion of mucins by HT29-MTX cells, it is possible to see that claudin-1 and *E*-cadherin are expressed in all the models at the different time points (Figures 6B,C). Comparing the 2D Caco-2 model at different days, there is a higher expression of the proteins on day 21 comparing to day 14. Nevertheless, when comparing the 2D Coculture models, although the expression of *E*-cadherin seems higher at day 21, the same does not happen with the expression of claudin-1, which seems lower. Regarding the 3D models, the expression of *E*-cadherin is in the middle of the expression of the 2D models, whether for claudin-1, expression seems to be similar to the 2D models at 21 days.

**FIGURE 7:**
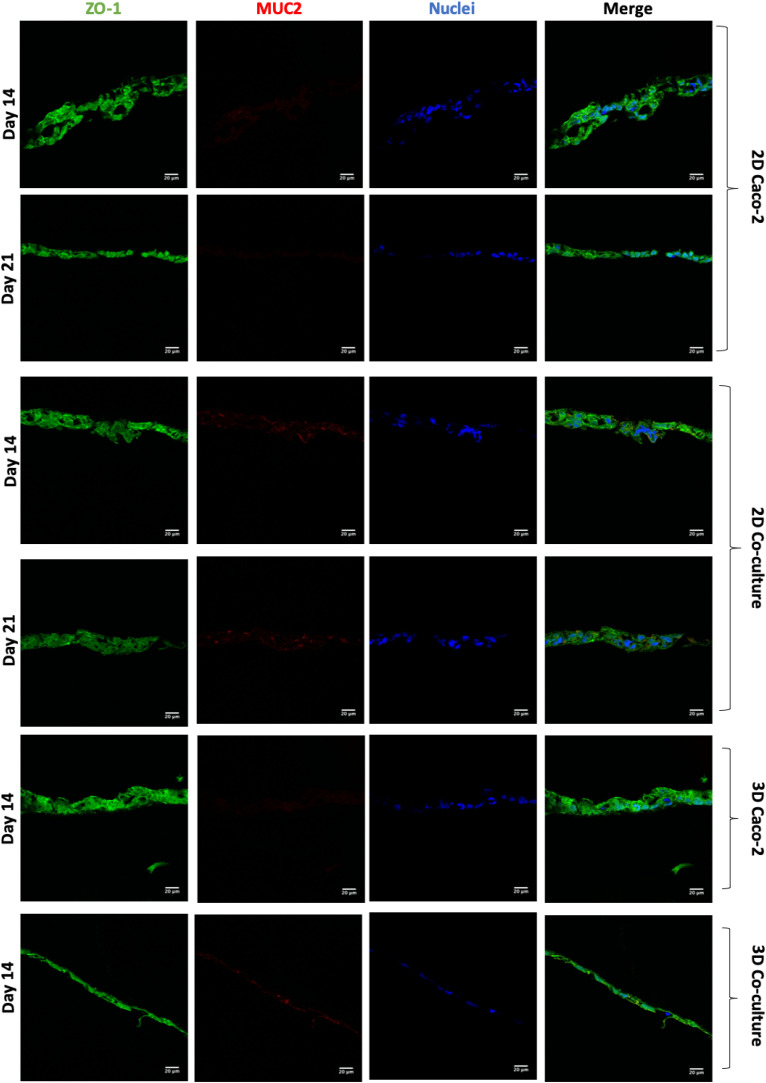
Assessment and comparison of the tight junctions’ protein ZO-1 and the mucin MUC2 in the different models at different time points. ZO-1 was labeled with Alexa-Fluor 488 (green), and MUC2 was labeled with Alexa Fluor 594 (red). The nucleus was counterstained with 4′,6-diamidino-2-phenylindole (DAPI) (blue).

Regarding the expression of the TJ protein ZO-1 (Figure 7), it is possible to observe that all the models have a high expression of this protein, and there are no visible differences between the models. Regarding the expression of MUC2 (Figure 7), as it was expected, this mucin is only secreted when HT29-MTX cells are present in the model, since these are mucus-secreting cells, unlike Caco-2 cells.

### Permeability Assay With Fluorescein Isothiocyanate–Dextran 4 kDa

**FIGURE 8:**
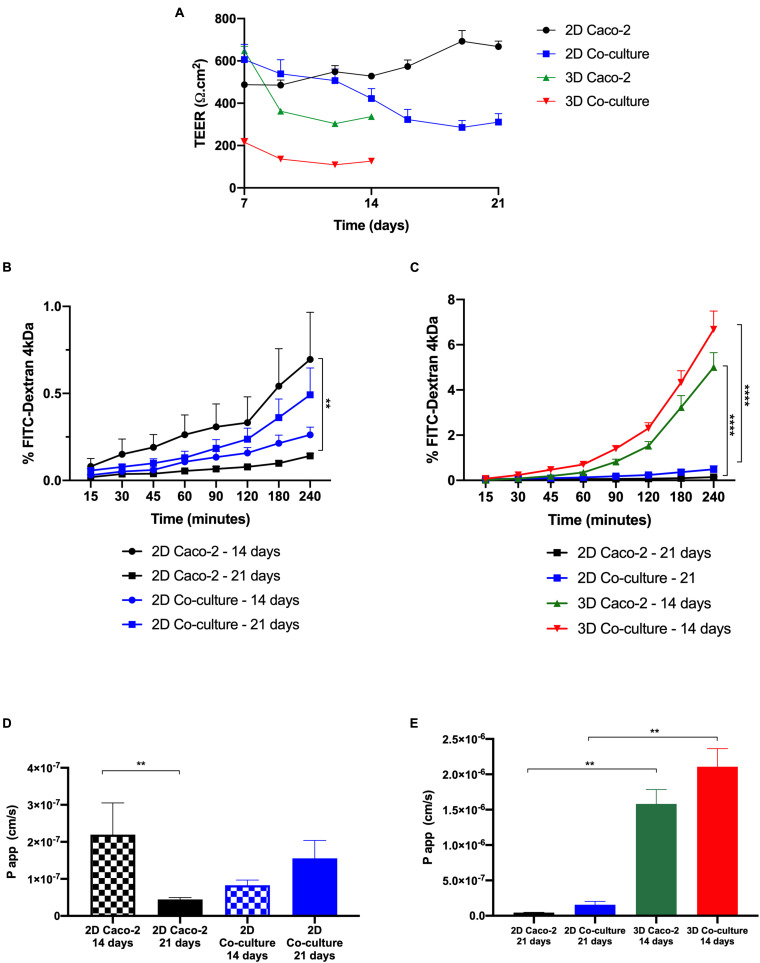
Comparison between two-dimensional (2D) and 3D models regarding transepithelial electrical resistance (TEER) values and permeability. **(A)** TEER values of the models over time. Comparison of the cumulative transport of fluorescein isothiocyanate (FITC)–Dextran across the **(B)** 2D models after 14 vs. 21 days in culture and **(C)** 3D models after 14 days in culture vs. 2D models after 21 days in culture. Comparison of Papp values of **(D)** 2D models after 14 vs. 21 days in culture and **(E)** 3D models after 14 days in culture vs. 2D models after 21 days in culture. Results are the average of triplicates (except for permeability of 3D Caco-2, which is *n* = 2), and bars represent the standard error deviation (SEM). Statistical differences *p* < 0.05 are denoted by *, *p* < 0.01 are denoted by **, and *p* < 0.0001 are denoted by ****.

The barrier integrity of the intestinal models was accessed by TEER measurements along the time in culture (Figure 8A). As expected, the 2D Caco-2 model was the one with higher TEER values, around 800 Ω.cm^2^. The addition of HT29-MTX cells to the model renders a decrease in TEER to approximately 350 Ω.cm^2^. Regarding the 3D models, both models present lower TEER values than their 2D analogs. As it happens in the 2D models, in 3D, the Caco-2 model presents higher TEER values than those in the 3D Coculture.

Regarding the permeability outcomes, it was observed that all models show a similar tendency of increased permeability with time. It can be observed in Figures 8B,D that regarding the 2D Caco-2 model, there are significant differences between permeability values at days 14 and 21, being that permeability is higher at day 14 than 21. Regarding the 2D Coculture model, the opposite occurs. When comparing the permeability of the 2D models at day 21, that is what is described in the literature as the proper time for the cells to be in culture and form a confluent and polarized monolayer, with the 3D models at day 14 (Figures 8C,E), it is possible to observe that 3D models present higher permeability, when comparing to their 2D analogs, being that the 3D Coculture model is the model that presents the greatest permeability.

## Discussion

In the present study, a new 3D *in vitro* intestinal model was developed. The main purpose of the study was to obtain a more physiologically relevant intestinal *in vitro* model that could render more accurate results when testing the absorption of paracellular compounds in the gut and in a faster way. To obtain this model, a collagen layer with HIFs embedded was optimized in order to mimic the intestinal lamina propria, which is the substrate where the epithelial cells sit in the small intestine (Powell et al., 2011). Any active compound to reach the systemic circulation must cross the epithelium, but also the lamina propria, which is, mostly, neglected in the *in vitro* screening of drug absorption. Besides, the lamina propria provides 3D support to the epithelium and gives important cues to the cells, influencing their behavior (Griffith and Swartz, 2006; Castano et al., 2019). Since it is well established that fibroblasts have the ability to degrade and contract collagen (Zhu et al., 2001; Branco da Cunha et al., 2014), a high collagen concentration of 5 mg/ml was used and different HIF seeding densities were embedded in the hydrogels to access their behavior. This high concentration of collagen was also based on the concentration in native tissues, which is normally above 5 mg/ml, despite most 3D models that are developed use lower concentrations, not mimicking the native tissue (Antoine et al., 2014). It was observed that even with this high concentration of collagen, fibroblasts were able to contract the gel and that higher initial amounts of fibroblasts led to higher contraction and degradation of the collagen disks. Since an intact disk covering the entire area of the Transwell^®^ insert is needed, otherwise it would not be possible to perform reliable permeability assays, an optimization of the layer had to be performed. With an optimized collagen concentration of 6 mg/ml and an HIF seeding density of 1 × 10^5^ cells/ml, the disks’ rheological properties were evaluated, indicating the disks are soft matrices and rheological values are within normal values for the intestinal lamina propria, whose elastic modulus is around 0.5 and 1 kPa (Stidham et al., 2011; Stewart et al., 2018; Costa and Ahluwalia, 2019). Nevertheless, comparisons with human values or values found in the literature are not straightforward. Using different equipment, e.g., a rheometer or a nanoindentation tester, or even using the same equipment but changing the geometry, introduces variability to the results, making it hard to compare with the existing literature. Regarding the hydrogels’ inner structure, it was possible to observe highly porous structures, as it was expected, since hydrogels are composed mainly of water, enabling the survival and proliferation of fibroblasts (Ahmed, 2015).

The metabolic activity of the cells inside the gels along the time in culture was assessed. Although it is important that fibroblasts do not contract the gel, it is also very important that they are healthy and metabolically active inside the gels, obtaining a compromise between fibroblast proliferation and disk integrity. During the time in culture, the metabolic activity of fibroblasts inside the gels significantly increased every week. Besides metabolic activity, it is also important to understand if cells present their physiological elongated shape. When fibroblasts are surrounded by stiff matrices, they are not able to spread, staying in a round shape, which is not representative of what happens in the human body (Branco da Cunha et al., 2014). Regarding the morphology and ECM component secretion of fibroblasts, it was possible to observe that fibroblasts presented an elongated shape when entrapped in the collagen matrix and were able to secrete fibronectin and laminin, shown to be key players in the remodeling of the matrix. After 14 days in culture, the disks are no longer comprised of only type I collagen from rat tail, but human fibronectin and laminin, secreted by the fibroblasts. This indicates that fibroblasts are able to degrade the collagen and proliferate, secreting their own matrix, remodeling their surrounding environment, which is important for cell survival (Roulis and Flavell, 2016).

It is well known that cells have the ability to communicate with each other, this phenomenon being regarded as cellular crosstalk (Geiger et al., 2001). It was, then, investigated whether epithelial cells on top of the collagen gels could exert some effect on the fibroblasts inside the collagen gels. When epithelial cells were added to the cultures, a higher number of fibroblasts inside the disks were observed, which seem to indicate that epithelial cells enhance the proliferation of stromal cells inside the collagen matrix. The secretion of ECM components, such as fibronectin and laminin, was also increased when this crosstalk occurred, which can be explained by the fact that epithelial adhesions with the ECM are most commonly mediated by integrins, and the extracellular ligands that anchor these adhesions include fibronectin (Geiger et al., 2001). There was also a higher number of fibroblasts when only Caco-2 cells were seeded on top, being that the addition of HT29-MTX seemed to have an inhibitory effect on the crosstalk between Caco-2 and the fibroblasts. Besides being visible by the higher number of fibroblasts and fibronectin deposition, this is also supported by the fact that 3D Caco-2 models contracted after 16 days in culture, whether 3D Coculture models maintained their integrity for 21 days, meaning that fibroblasts of the 3D Caco-2 models were more contractile than the ones of the 3D Coculture model.

Before performing the permeability assays with the 3D models at day 14, the establishment of an intact monolayer was confirmed, since an intact barrier is crucial to perform reliable permeability experiments. Since TEER values of the models were lower in the 3D comparing to the 2D models, H&E and EpCAM stainings were performed to observe the cellular barrier. It was observed by both methods that at 14 days, the monolayer of the 3D models is already completely formed, as well as the monolayer of the 2D models, and no visible differences in the 2D models are observed between days 14 and 21. Indeed, it is described that when cells are seeded on top of biological matrices, instead of artificial ones like the Transwell^®^ membrane, the TEER decreases (Li et al., 2013). This decrease in TEER is usually not associated with barrier integrity, but with the tightness of the TJs (Li et al., 2013; Yi et al., 2017). When cells are seeded on top of 3D biological matrices, they tend to show a more *in vivo* behavior, not forming such tight TJs and becoming more similar to what is observed in the intestinal epithelium, where TEER values are around 30 Ω (Legen et al., 2005). The decrease in the TEER values is likely related to the TJs and not barrier integrity. Claudins are able to bind to ZO, through a PDZ-binding motif. Claudins are the major determinant of barrier junction. Intestinal claudins form two different classes: sealing and pore forming. Claudin-1 belongs to the sealing class, and if its expression is increased, this results in a tighter barrier, which restricts the movement of luminal contents through the paracellular route (Hamazaki et al., 2002; Shen et al., 2011). Regarding ZO-1, besides binding to claudins and other transmembrane proteins, is also able to interact with actin cytoskeleton and its associated proteins through the C-terminal region, which is very important to maintain TJ formation and function (Hamazaki et al., 2002; Lee, 2015; Lee et al., 2018). The presence of cadherins, which are responsible for cellular adhesion, was also assessed. It was possible to observe that for the 2D models, the expression of claudin-1 and *E*-cadherin seems to be higher at day 21 than at day 14, which is in accordance to TEER values. Nevertheless, the expression of ZO-1 seems to be similar in the 2D Caco-2 models at days 14 and 21, whether for the Caco-2/HT29-MTX 2D Coculture, expression is higher at day 14, which could be explained by the higher presence of HT29-MTX cells at day 21, since they have 1 more week to proliferate and increase their number in the model, and since these cells do not possess so many TJ proteins as the Caco-2 cells, its expression is lower. Regarding the expression of TJ proteins by the 3D models, it was observed that both claudin-1 and ZO-1 were expressed to the same extent as observed on the 2D models, leading to the conclusion that TJs are present and are not downregulated in the models. The presence of the MUC2 mucin was also evaluated and confirmed the ability of the HT29-MTX as mucus-producing cells, resembling the intestinal goblet cells.

After it was confirmed that 3D models at 14 days of culture presented an intact barrier, permeability studies were performed. First, as control, permeability assays with FITC-Dextran 4 kDa in 2D models at days 14 and 21 were performed. A significant difference was observed between the 2D Caco-2 model at day 14 and day 21, with permeability being higher at day 14. This can be explained by the fact that at day 14, the Caco-2 monolayer in the 2D model does not possess such tight TJs, which makes the compound pass more easily. This is actually sustained by the expression of claudin-1, with higher expression at day 21, comparing to day 14. Since it is described that the Caco-2 model is not a suitable model to study the permeability of paracellular compounds because of the tightness of the barrier, making permeability underestimated, maybe it would be better to perform permeability assays at day 14 when using the Caco-2 model to study paracellular transport (Tavelin et al., 2003). Nevertheless, it is important to say that permeability was still low on the 2D Caco-2 model at day 14, and that even with the addition of HT29-MTX cells, which are able to decrease the TEER of the barrier, values of absorption of dextran remained very low (Antunes et al., 2013). Regarding the 2D coculture, the opposite occurs, and it is possible to observe that permeability is higher at day 21 than at day 14. We hypothesize that this is related to the number of HT29-MTX cells that increases with time in culture. Since these cells do not possess such tight TJs, a higher number of these cells in the model may improve the passage of compounds paracellularly.

Considering the widely established protocols for 2D Caco-2 and Coculture models for 21 days in culture, the permeability of these models with the values obtained for the 3D models after 14 days was compared (Hidalgo et al., 1989; Delie and Rubas, 1997). When relating the 2D with the 3D models, it was possible to see that the permeability of FITC-Dextran was significantly higher in the 3D models. These results are in accordance with the TEER values, since the 3D models present lower TEER values than the 2D models, which means the compound can pass more easily between the cells. Nevertheless, when comparing the TEER values of the 3D Caco-2 model at day 14 with the values of the 2D Coculture model at day 21, values are similar and around 300 Ω.cm^2^. However, when it comes to permeability, the values are completely different, being that permeability on the 3D Caco-2 model is five times higher than in the 2D Coculture model. This discrepancy indicates that permeability is not just merely related to the TEER values but most likely to the cells themselves and the fact that a 3D layer that mimics the *in vivo* environment is present that can have a huge impact on cellular behavior, as already stated. In fact, permeability phenomenon may be regulated not only by the fact that cells are seeded on top of the collagen layer but also by the crosstalk between the epithelial and stromal cells that we have seen have an important role in the remodeling of the matrix (Geiger et al., 2001). It is likely that this crosstalk also influences the behavior of cells regarding the permeability of compounds. This correlates with the expression of TJs. When comparing the expression of claudin-1 and ZO-1, which are TJ proteins, it seems that the expression in the 3D models at day 14 is similar to the expression of the 2D models at day 21. It is expected that the TEER values are not only regulated by the TJ expression, but other mechanisms may have a role. Besides, it is also a proof that the absorption of the paracellular compounds does not depend merely on TJ expression and barrier tightness but that the cells are in a more *in vivo* state may influence their behavior at other levels, and this may affect the permeability of the compounds. Besides, although it was not tested in the present work, we believe that this model can be suitable to test the permeability of not only paracellular compounds but also compounds that cross the intestinal barrier transcellularly. In fact, it has been reported that cells in 3D models may present a more relevant expression of drug transporters, like P-glycoprotein (P-gp), an efflux transporter that can limit the absorption of several compounds, which can have a great impact on permeability (Li et al., 2013; Yi et al., 2017). So, this model should be further studied and its potential to be used as a model to evaluate the permeability of transcellular compounds should be assessed, since it would bring enormous added value to the model.

## Conclusion

In the present work, a novel 3D intestinal model was established, reproducing, in a more physiological way, the architecture and composition of the human small intestine. It was observed that fibroblasts embedded in collagen, mimicking the intestinal lamina propria, had the capability of producing ECM components, remodeling the surrounding environment and had a pivotal role in the behavior of epithelial cells. The 3D models were used for permeability assays after 14 days of culture, since it was observed that the epithelial layer was already formed and the expression of claudin-1 was similar to that of the 2D models at day 21. Besides, although TEER values presented differences between the 2D and 3D models, the expression of TJs (claudin-1 and ZO-1) and adherens junctions was similar between them. The permeability of FITC-Dextran 4 kDa, a hydrophilic compound that is known to cross the epithelium by the paracellular route, was significantly higher in the 3D models. We believe that these results are important, since it is known that paracellular permeability in the Caco-2 model is underestimated, since these cells resemble more the colonic epithelium. These results show that when cells are in a more physiological configuration and sensing a biological 3D matrix, instead of the Transell insert membrane, there is an effect on the cells, making them behave more similarly to what is observed *in vivo*. In fact, we believe that a crosstalk between epithelial and stromal cells occurs during the maturation of the model, since there is an enhancement on HIF contraction capability when Caco-2 cells are seeded on top of the collagen gels with fibroblasts that is likely regulated by the secretion of cytokines and growth factors by the cells. In conclusion, a new *in vitro* intestinal model is herein proposed to better and faster predict the permeability of paracellular compounds, helping in bridging the gap between *in vitro* models and animal testing and contributing to the 3Rs policy.

## Data Availability Statement

The raw data supporting the conclusions of this article will be made available by the authors, without undue reservation, to any qualified researcher.

## Author Contributions

MM performed the experimental work. BS, CB, and EM provided the scientific guidance. All authors contributed to the writing of the manuscript.

## Conflict of Interest

The authors declare that the research was conducted in the absence of any commercial or financial relationships that could be construed as a potential conflict of interest.
