# Supplementary material for: Development of an Improved 3D in vitro Intestinal Model to Perform Permeability Studies of Paracellular Compounds
Source: Front Bioeng Biotechnol. 2020 Sep 17;8:524018. doi: 10.3389/fbioe.2020.524018 (PMC7527803; doi:10.3389/fbioe.2020.524018)
Supplement: Supplementary file 1 [file Image_1.pdf]

Supplementary materials

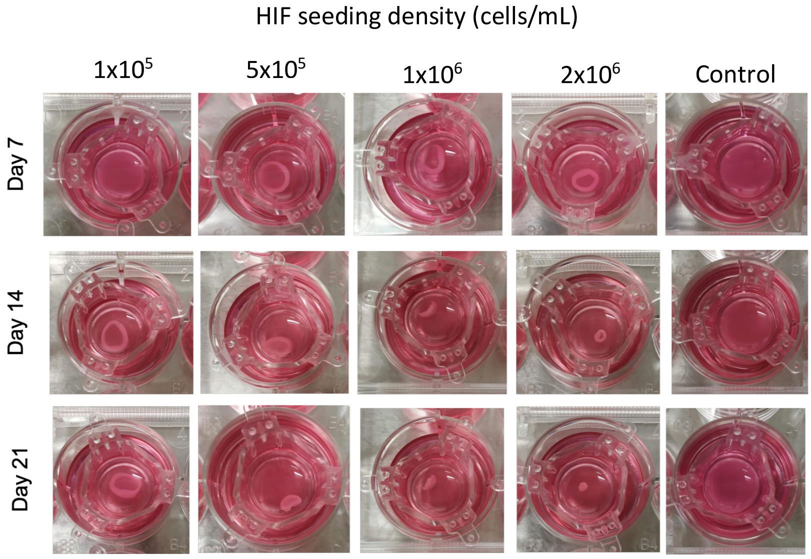

Figure 1 – First experiment with collagen to determine the optimal concentration of HIF. Different HIF seeding densities where embedded in the disks with a collagen concentration of 5 mg/mL. The behavior of the disks was accessed during 21 days and it was observed that higher amounts of fibroblasts led to higher collagen contraction.
